# Study of C-reactive protein, procalcitonin, and immunocyte ratios in 194 patients with sepsis

**DOI:** 10.1186/s12873-021-00477-5

**Published:** 2021-07-07

**Authors:** Tian Tian, Bing Wei, Junyu Wang

**Affiliations:** Department of Emergency Medicine, Beijing Chaoyang Hospital Jingxi Branch, Capital Medical University, No.5 Jingyuan Road, Shijingshan, Beijing, 100043 China

**Keywords:** Sepsis, C-reactive protein, Procalcitonin, Immunocyte, Diagnosis

## Abstract

**Background:**

Evidence suggests that C-reactive protein (CRP), procalcitonin (PCT), and immune cells can predict sepsis severity in adult patients. However, the specific values of these indicators are not consistent in predicting prognosis.

**Methods:**

A retrospective study analyzed the medical records of 194 patients based on the concept of sepsis in 2016 (Sepsis 3.0) from January 2017 to December 2019. A comparative analysis of inflammatory factors associated with patients in the sepsis survival and the non-survival group was performed. The concentrations of CRP and PCT, neutrophil-to-lymphocyte ratio (NLR), monocyte-to-lymphocyte ratio (MLR), and platelet-to-lymphocyte ratio (PLR) were measured. ROC curve was used to assess the diagnosis and analysis of the selected indices of sepsis. According to each index’s cut-off value of the ROC curve, the patients were divided into two groups, and the prognosis was calculated.

**Results:**

Among the 194 patients, 32 died (16.49%), the median age of the patients was 79 (66.0, 83.3) years, and 118 were male (60.8%). Analysis of related inflammatory indicators showed that CRP, NLR, MLR, PLR, and CRP*PCT in the non-survival group were statistically higher than those in the survival group (all *p* values were < 0.05). Regression analysis showed that PCT, CRP, NLR, PLR, and CRP*PCT were all independent prognostic factors for patients. The ROC curve results showed that CRP*PCT had the best diagnostic value (AUC = 0.915). The cut-off values of PCT, CRP, NLR, PLR, MLR, and CRP*PCT were 0.25 ng/mL, 85.00 mg/L, 8.66, 275.51, 0.74%, and 5.85 (mg/L)^2^, respectively. Kaplan-Meier survival estimate showed that patient prognosis between the CRP, PCT, NLR, PLR, and CRP*PCT was statistically different (all values *P* < 0.05, respectively). However, there was no statistically significant difference in gender and MLR (all values *P* > 0.05, respectively), grouping based on diagnostic cut-off values.

**Conclusions:**

In this study, inflammation-related markers PCT, CRP, NLR, MLR, PLR, and CRP*PCT can be used as independent risk factors affecting the prognosis of patients with sepsis. Furthermore, except for MRL, these indicators have cut-off values for predicting patient death.

## Background

Sepsis is an acute syndrome triggered by an infection that can lead to severe sepsis or septic shock [1–3]. There are more than 19 million severe sepsis cases worldwide each year [4]. Severe sepsis is a condition of a high mortality rate with an estimated yearly worldwide prevalence exceeding 19 million. The detection of sepsis-related indicators can evaluate the patient’s severity and then assess the patient’s condition and adjuvant treatment.

Procalcitonin (PCT) and C-reactive protein (CRP) are the main biomarkers for diagnosing sepsis. PCT concentration in the blood of healthy people is deficient, more diminutive than 0.05 ng/mL [5]. In the state of inflammation, especially bacterial infection or sepsis, various tissues and cell types of the body can produce PCT and release it into the blood [6]. PCT levels increase rapidly in the early phase of the systemic inflammatory response caused by a bacterial infection, which can be detected within 2–3 h, and reaches a peak about 12–24 h after infection. Simultaneously, the PCT level can effectively reflect the severity of bacterial infection in patients, and its concentration is positively correlated with the severity of systemic bacterial infection [7, 8]. CRP is an acute-phase protein biomarker widely used in clinical practice. The liver mainly synthesizes CRP under the stimulation of interleukin-6 [9]. Clinically, CRP has good value in screening critically ill patients, diagnosing infections, and evaluating patient response to antibiotic treatment [10, 11]. As a sensitive indicator of inflammation, CPR detection is quick and convenient, and CRP increase is positively correlated with the severity of infection or inflammation [12, 13].

Systemic immune-inflammation index (SII) can be used to evaluate the severity of sepsis in patients, including the percentage of neutrophil to lymphocyte count (NLR), the platelet to lymphocyte ratio (PLR), the monocyte to lymphocyte count (MLR), and the mean platelet volume to platelet count (MPV/PC) [14–17]. The use of combined detection of multiple indicators might potentially be the future development trend, improving early diagnosis and prognostic value of infectious diseases.

This study compared PCT and CRP expression levels and related immune-inflammatory cell ratios in the non-survival group and the survival group. This study further evaluated sepsis diagnosis level by different indicators and provided a comprehensive analysis and research of patients’ complete diagnosis.

## Methods

### Collection of patient baseline data

In this study, a retrospective analysis was carried out to collect the medical records of 194 patients with sepsis treated at the Intensive Care Unit (ICU) ward of our hospital from January 1, 2017, to December 29, 2019. This study was approved by Beijing Chaoyang Hospital’s ethics committee (No. 2016-KE-143). Written informed consent by patients or their family members was obtained, and patient confidentiality was strictly maintained.

### Diagnosis and inclusion and exclusion criteria of patients with sepsis

The inclusion criteria for all patients were based on the concept of sepsis in 2016 (Sepsis 3.0) [18–21]. Sepsis severity defined as when Sequential Organ Failure Assessment (SOFA) score was ≥2; the septic shock is defined as a specific subtype of sepsis. Acute Physiology and Chronic Health Evaluation (APACHE2) scores were used to assist in diagnosing sepsis. The study inclusion criteria were age ≥ 18 years, CRP test within 6 h of arrival at the ICU ward, and original record. The exclusion criteria were unknown infection, ICU stays < 72 h, incomplete clinical data, or second ICU admission.

### Collection and detection methods of blood samples

Routine blood tests were performed on the day of admission. The patient’s blood samples were processed as soon as possible after collection, and the experiment was carried out as quickly as possible after extraction. EDTA-K2 anticoagulant blood collection tubes were used in all patients to collect about 2 ml blood after an overnight fast and sent to the laboratory for testing. If the test cannot be performed immediately, the specimen was stored at − 20 °C, but repeated freezing and thawing were avoided. The KX-21 automatic blood cell analyzer (Nishimikang, Japan) was used obtain white blood cell count, neutrophil ratio, lymphocyte ratio, monocyte ratio, red blood cell count, hemoglobin, hematocrit, and platelet count in the venous blood samples from the patients. Different rates of immunocytes were used in the diagnostic study of patients with sepsis, including the ratio of neutrophil to lymphocyte count (NLR), the ratio of platelet to lymphocyte ratio (PLR), the ratio of monocyte to lymphocyte count (MLR), and the ratio of mean platelet volume to platelet count (MPV/PC).

### PCT detection

PCT was detected by the immunosandwich method using Mérieux’s mini VIDAS automatic fluorescence immunoassay analyzer. The reagent was the company’s VIDAS BRAHMS PCT quantitative determination kit, and the normal reference range was < 0.5 μg/l.

### CRP detection

Serum samples from both groups to be tested were assayed using CRP antibody (465131) provided by Beckman Coulter. The expression level of CRP was measured by the turbidimetric method with a reference range of standard values from 0 to 8 mg/L.

### Statistical methods

All data processing in this study used statistical software SPSS 25.0 (IBM) and MedCalc (Version 92.1.0, Belgium). Numerical data were tested using the chi-square test. Normally distributed data were expressed in mean ± standard deviation. Comparison between two groups used Student’s t-test, and the comparison of three or more data groups was done by variance test. The Mann-Whitney test was used for the pairwise calculation of non-normally distributed data. The Kruskal-Wallis method was used for multiple group comparison. Logistic regression analysis was carried out. The diagnostic tests and combined diagnostic tests were used to analyze patients’ diagnostic indicators in the sepsis survival group and the non-survival group. The ROC curve was drawn to calculate sensitivity, specificity, the Youden index, and the area under the ROC curve. The Kaplan-Meier was used for estimating the survival function. When *P* < 0.05, the difference was considered to be statistically significant.

## Results

### Patient baseline data

A total of 194 patients with sepsis were included in the study. Among the patients, 32 died during hospitalization, and the mortality rate was 16.49%. Among the patients, the median age was 79 (66.0, 83.3) years, and 118 patients were male (60.8%). Patient information was collected from January 1, 2017, to December 29, 2019. The majority of the patients with sepsis were due to lung infection (167/194, 86.08%) and combined abdominal (6/194, 3.09%) and urinary tract infection (5/194, 2.58%). The second infection factor was abdominal infection (16/194, 8.25%). Among the patients, half of the deaths were caused by stroke (16/32, 50.00%). The median length of hospital stay in the survival group was 7.0 (5.8, 11.0), and the median length of hospital stay in the non-survival group was 8.5 (6.0, 13.8). There was no significant difference between the two groups (*P* = 0.543). Totally 118 cases were male, accounting for 60.82%; there was no difference in death between male and female patients (*P* = 0.854). The median age of the survival group was 78.0 years (63.5, 83.0), and the median age of the non-survival group was 81 years (76.0, 86.5). Still, the difference between the two groups was not statistically significant (*P* = 0.063). More detailed results are shown in Table 1.

**Table 1:** Patient’s baseline data

Grouping	Survival group (162)	Death group (32)	Z/X^2^	P
Follow-up Time	7.0 (5.8, 11.0)	8.5 (6.0, 13.8)	−0.609	0.543
Gender				
Male	99	19	0.034	0.854
Female	63	13		
Age (years)	78 (63.5, 83)	81 (76, 86.5)	−1.858	0.063
Shock				
Yes	9 (5.56%)	16 (50.00%)		< **0.001**
No	153 (94.44%)	16 (50.00%)		
Site of infection				
Lung	142 (87.65%)	25 (78.13%)		< **0.001**
Abdomen	12 (7.41%)	4 (12.50%)		
Lung+ Abdomen	4 (2.47%)	2 (6.25%)		
Lung + Urinary system	4 (2.47%)	1 (3.12%)		
PCT	0.050 (0.050, 0.093)	1.095 (0.143, 4.815)	−6.841	< **0.001**
CRP	12.000 (10.000, 33.000)	109.094 ± 69.362	−5.696	< **0.001**
NLR	5.468 (3.473, 9.503)	12.904 (7.156, 26.910)	2.311	< **0.001**
MLR	0.426 (0.303, 0.620)	0.713 ± 0.458	−2.898	**0.004**
PLR	168.729 (123.896, 264.698)	326.042 (139.877, 598.006)	−3.153	**0.002**
MPV/PC	0.046 (0.036, 0.061)	0.049 (0.030, 0.068)	−0.160	0.873
CRP*PCT	1.000 (0.600, 4.325)	100.060 (11.000, 408.840)	−7.432	< **0.001**
SOFA	4.0 (3.0, 6.0)	5.5 (4.0, 9.0)	−2.862	**0.004**
APACHE2	18.0 (13.0, 26.3)	36.0 (32.0, 39.0)	−6.998	< **0.001**

### Analysis of related inflammatory indicators

Current studies have found that various blood markers can reflect the severity of sepsis. PCT and CRP are the main diagnostic biomarkers of sepsis. The analysis of related inflammatory indexes of the two groups showed that the value of PCT in the non-survival group was significantly higher than that in the survival group (*P* < 0.001); the value of CRP in the non-survival group was significantly higher than that in the survival group (*P* < 0.001). The value of NLR in the non-survival group was significantly higher than that of the survival group (*P* < 0.001). The value of MLR in the non-survival group was significantly higher than survival group (*P* < 0.001); the value of PLR in the non-survival group was significantly higher than that in the survival group (*P* < 0.001); the CRP*PCT in the non-survival group The value was significantly higher than that of the survival group (*P* < 0.001) while the MPV/PC ratio was not statistically different between the two groups (*P* = 0.873). The detailed results are shown in Table 1.

### Regression analysis

The univariate regression analysis showed that age, CRP value, and NLR were inversely associated with the patient’s survival (Table 2). The multivariate regression analysis showed that under the premise of excluding confounding factors, PCT, CRP, NLR, PLR, and CRP*PCT were all independent prognostic factors of sepsis (Table 3).

**Table 2:** The results of univariate regression analysis

Groups		B	SE	Wald	DF	Sig.	Exp(B)
Step 1^a^	Sex (1)	0.215	0.642	0.113	1	0.737	1.240
	Age	−0.061	0.027	5.262	1	**0.022**	0.941
	PCT	−0.462	0.246	3.519	1	0.061	0.630
	CRP	−0.015	0.006	6.377	1	**0.012**	0.985
	NLR	−0.103	0.047	4.696	1	**0.030**	0.903
	MLR	0.577	0.763	0.572	1	0.449	1.780
	PLR	0.000	0.002	0.024	1	0.876	1.000
	MPV/PC	−9.298	8.166	1.296	1	0.255	<0.001
	CRP*PCT	−0.007	0.006	1.353	1	0.245	0.993

a. Variable entered in step 1:Sex, age, PCT, CRP, NLR, MLR, PLR, MPVPC, and CRP*PCT

SE: Standard error; DF: degree of freedom; Sig.: Significance

**Table 3:** The results of multivariate regression analysis

Groups		B	SE	Wald	DF	Sig.	Exp(B)
Step 1^a^	Sex (1)	0.072	0.394	0.034	1	0.854	1.075
	Age	−0.027	0.016	2.653	1	0.103	0.974
	PCT	−0.634	0.158	16.032	1	**< 0.001**	0.530
	CRP	−0.018	0.003	32.155	1	**< 0.001**	0.982
	NLR	−0.100	0.023	19.402	1	**< 0.001**	0.905
	MLR	−0.885	0.392	5.109	1	**0.024**	0.413
	PLR	−0.003	0.001	10.121	1	**0.001**	0.997
	MPV/PC	−5.380	3.412	2.485	1	0.115	0.005
	CRP*PCT	−0.023	0.006	16.362	1	**< 0.001**	0.977

a. Variable entered in step 1:Sex, age, PCT, CRP, NLR, MLR, PLR, MPVPC, and CRP*PCT

### Diagnostic value of inflammation indicators

**Fig. 1:**
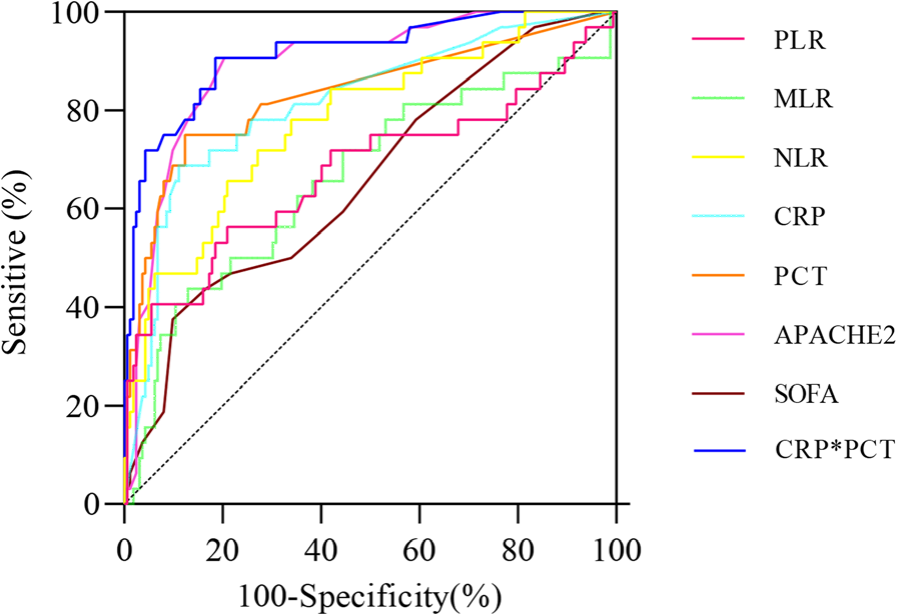
The ROC curve of C-reactive protein (CRP), procalcitonin (PCT), and different immunocyte ratios. NLR: the percentage of neutrophil to lymphocyte count; PLR: the percentage of platelet to lymphocyte ratio; MLR: the ratio of monocyte to lymphocyte count; MPV/PC: the ratio of mean platelet volume to platelet count; SOFA: Sequential Organ Failure Assessment; APACHE2: Acute Physiology and Chronic Health Evaluation

In the diagnostic analysis of the six index values selected by multi-factor screening, the ROC curve results showed that CRP*PCT had the best diagnostic value (sensitivity: 90.62%, specificity: 81.48%; Z = 13.910, *P* < 0.001), the AUC area was 0.915, and the Youden index was 0.721. The diagnostic value of APACHE2 was inferior to CRP*PCT (sensitivity: 90.62%, specificity: 79.63%; Z = 12.930, *P* < 0.001), with an AUC area of 0.891, and the Youden index was 0.703. The diagnostic value had different degrees of emphasis among other indicators. More detailed results are shown in Table 4 and Fig. 1. The cut-off values of PCT, CRP, NLR, PLR, MLR, CRP*PCT, SOFA, and APACHE2 were 0.25 ng/mL, 85.00 mg/L, 8.66, 275.51, 0.74%, 5.85 (mg/L)^2^, 8, and 29, respectively.

**Table 4:** Diagnostic parameter results of different factors

Variable	AUC	SE	95% CI	Sensitive (%)	Specificity (%)	Yuden Index (%)	Cut-off	Z	P
CRP	0.815	0.044	0.753–0.867	68.75	88.89	0.576	85.00 mg/L	7.109	**< 0.001**
CRP*PCT	0.915	0.030	0.866–0.950	90.62	81.48	0.721	5.85	13.910	**< 0.001**
MLR	0.663	0.059	0.591–0.729	43.75	87.04	0.308	0.74%	2.756	**0.006**
NLR	0.780	0.047	0.715–0.836	71.87	72.84	0.447	8.66%	5.983	**< 0.001**
PCT	0.833	0.044	0.773–0.883	75.00	87.65	0.627	0.25 ng/ml	7.531	**< 0.001**
PLR	0.677	0.063	0.606–0.742	56.25	79.01	0.353	275.51	2.808	**0.005**
SOFA	0.659	0.0534	0.587–0.725	37.50	90.12	0.2762	> 8	2.968	**0.003**
APACHE2	0.891	0.0303	0.839–0.931	90.62	79.63	0.703	> 29	12.930	**< 0.001**

### The patient’s prognosis

**Fig. 2:**
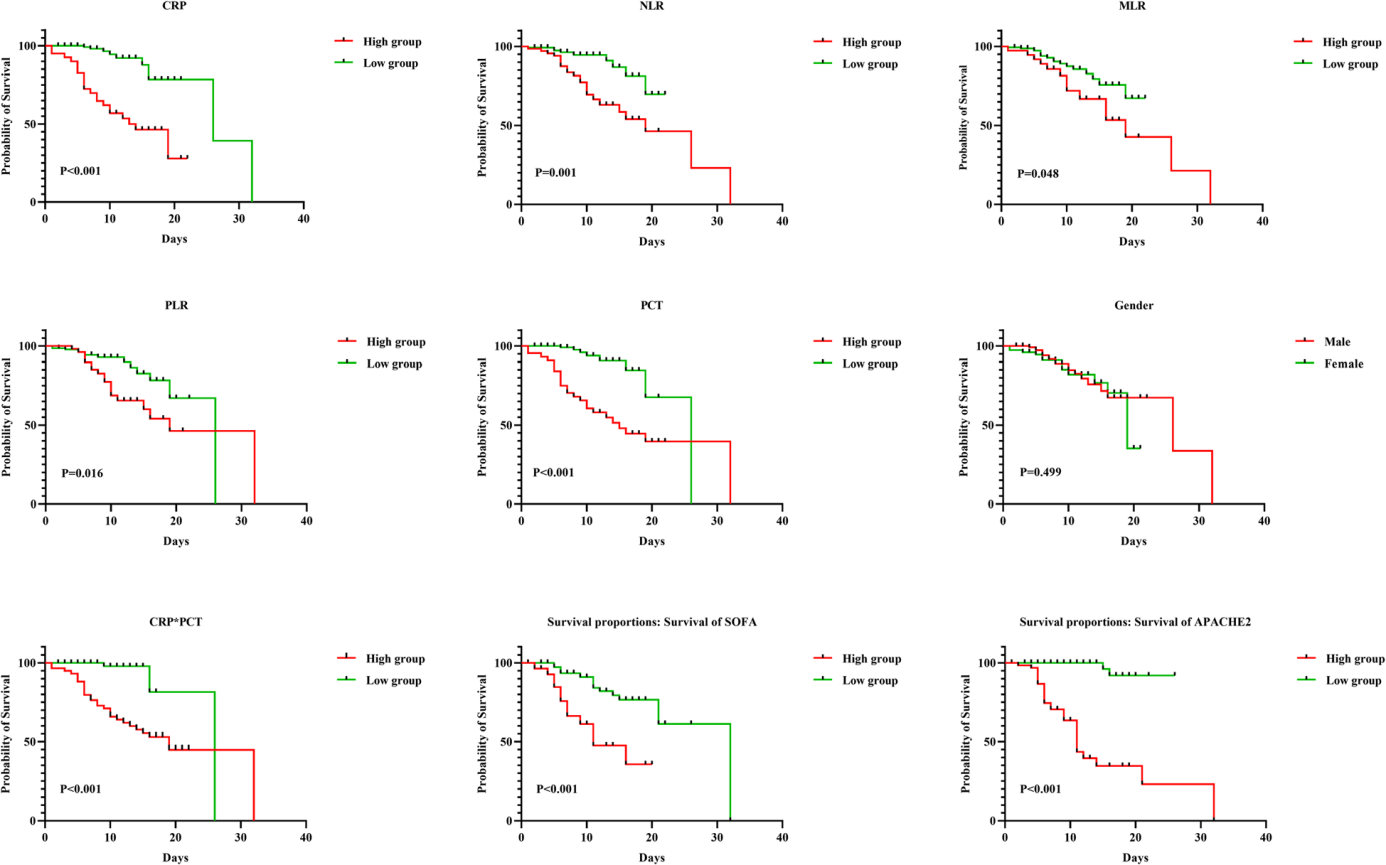
The prognosis of each factor (stratified by diagnostic cut-off value)

The patient’s prognosis showed no statistical difference in patients of different genders (*P* = 0.499) (Fig. 2). According to the cut-off value of the factor, the related inflammatory indicators were divided into two groups. Except for MLR, PCT, CRP, NLR, PLR, SOFA, APACHE2, and CRP*PCT were statistically significant independent predictors of mortality (all values of *P* < 0.05, respectively) (Fig. 2).

## Discussion

Sepsis is an inflammatory response syndrome caused by a wound infection. International epidemiological data suggests the fatality rate of sepsis patients has exceeded that of myocardial infarction and has become the leading cause of non-cardiac deaths in the ICU [22, 23]. Statistics show that approximately 44,000 people die from sepsis in the UK each year [24]. Therefore, an effective method to reduce the mortality of patients with sepsis is effective diagnostic methods. A total of 194 patients with sepsis were included in this retrospective study, 32 of whom died during hospitalization, with a mortality rate of 16.49%. A total of 178 patients had lung infections among the included patients, 16 patients had abdominal infections, and half of the patients who died had a stroke (16/32, 50.00%).

Both PCT and CRP are commonly used clinical markers of inflammation and used for differential diagnosis and monitoring of bacterial infectious diseases. CRP is closely related to infection and related to many factors in the diagnosis of sepsis. Therefore, CRP can be used as an essential auxiliary index for the diagnosis of sepsis [25]. A meta-analysis of 18 clinical trials confirmed that PCT could effectively assist in diagnosing sepsis [26]. Clinical trial data show that when the diagnostic value of PCT is 1.1 μg/L, both sensitivity and specificity for identifying sepsis are 71%. In this study, the diagnostic cut-off value showed that the prognosis of patients in the high expression group of CRP and PCT was poor than that of the low expression group (*P* < 0.05 in both). CRP and PCT values can be used as independent risk factors for the prognosis of patients with sepsis. Studies have shown that high CRP levels may be a risk factor for acute kidney injury in sepsis [27], but initial CRP cannot be used as an indicator to predict the prognosis of patients with sepsis [28]. An accurate diagnostic threshold is a fundamental guarantee to improve the sensitivity and specificity of sepsis diagnosis. The cut-off value of CRP to diagnose sepsis is 85.00 mg/L. Studies have shown that as a biomarker of sepsis, the level of PCT is closely related to acute kidney injury in sepsis. However, there are still some doubts about the diagnostic value of the initial PCT level as a prognostic indicator for sepsis patients [29]. When the diagnostic threshold is 0.25 ng/mL, PCT has the highest sensitivity and specificity in this group of critical patients [30]. Compared with CRP, PCT is more accurate for diagnosing patients with suspected bacterial infections. It is also more sensitive when distinguishing bacterial infections from non-infectious inflammation, indicating bacterial infections from viral infections, and reduce unnecessary blood cultures.

Generally, among the various indicators for diagnosing infection in patients with sepsis, PCT has a high specificity, but the sensitivity is average, while CRP has a high sensitivity but low specificity [31–33]. When PCT and CRP are used in combination, they can make up for each other’s shortcomings, improve the specificity and sensitivity of infection diagnosis, and allow early treatment decisions for clinicians. Combining two markers can improve the diagnostic accuracy for sepsis, such as the combination of traditional and new markers. However, there are few clinical studies on PCT and CRP to assess sepsis in critically ill patients. There is a lack of in-depth research materials. This study shows that although the specificity (88.89%) of CRP is higher than its sensitivity (68.75%), the combination of the CRP*PCT is of the best diagnostic value for sepsis among all diagnostic indicators index (sensitivity is 90.62%, specificity is 81.48%), which also has the same diagnostic trend as the study by Glas et al. [34].

Neutrophils are the first line of defence of innate immunity, and lymphocytes are special inflammatory mediators of adaptive immunity. Platelets are a non-specific first-line inflammation marker regulating endothelial permeability and recruiting granulocytes and macrophages [35, 36]. Many studies have shown that NLR and PLR can comprehensively reflect inflammation and the immune status of the body. The increase in the two values means an increase in inflammation [37, 38]. The NLR can be used to assess the severity of stress and systemic inflammation in critical patients. In this study, although NLR is an independent risk factor for the prognosis of patients with sepsis (*P* = 0.030), its diagnostic sensitivity (71.87%) and specificity (72.84%) for sepsis are average. The PLR can predict the inflammatory response of patients with sepsis. In this study, although PLR can be used as an independent risk factor for the prognosis of patients with sepsis (*P* = 0.001), it has a low sensitivity (56.25%) and broad specificity for the diagnosis of sepsis (79.01%). The MLR can reflect microglia activity and can be used as a peripheral marker of inflammation in the brain [39]. Although MLR can be used as an independent risk factor for the prognosis of patients with sepsis (*P* = 0.024), it has a low sensitivity (43.75%). The diagnostic cut-off value in this study showed that the prognosis of the high expression group of MLR, NLR, and PLR was poor than that of the low expression group (*P* < 0.05 in all). The increase of MPV/PC indicates extensive coagulation activation in the body, increased platelet consumption, and platelet dysfunction, which means potential bleeding tendency and increasing risk of adverse events. An increased bleeding tendency will aggravate the possibility of dissection rupture and the degree of organ ischemia so that these patients have a higher mortality rate [17]. Secondly, the elevated MPV/PC ratio represents the severity of the inflammatory response in the aortic wall because activated platelets have a pro-inflammatory effect through the interaction of platelets and leukocytes, and the inflammatory response worsens aortic damage and increases the possibility of rupture [40]. However, this study shows the MPV/PC ratio is not a determinant of death, and this ratio cannot be used for the diagnosis of sepsis.

In this study, the diagnostic cut-off showed that the prognosis of patients in the high expression group of SOFA and APACHE2 was poor than that of the low expression group (*P* < 0.05 in both). Therefore, we believe that the APACHEII score and SOFA score can better assess the severity of the condition and the prognosis of death in sepsis patients. The prognosis of patients between PCT, CRP, NLR, PLR, and CRP*PCT were also statistically different according to the cut-off value of the factor (*P* < 0.05 in all).

However, this study still has certain limitations, such as (1) it is a retrospective study, which makes it difficult to exclude confounding factors, which affect the final comparative survey of clinical results; (2) this is a small-sample single-centre study, and the conclusions still require confirmation by larger-scale clinical trials; (3) the initial plasma levels of inflammatory factors only reflect the intensity of inflammatory response at the onset of the disease and the severity of the disease at the time, the evaluation of the clinician’s condition has a warning effect, but cannot completely represent the final prognosis of the patients.

## Conclusion

In summary, PCT, CRP, NLR, MLR, PLR, and CRP*PCT can be used as independent risk factors affecting the prognosis of patients with sepsis. The use of cutoff values for each indicator can be well used by clinicians to take early action on the risk of patient death. The CRP*PCT has a high diagnostic value for sepsis patients, and other indicators can be used as an auxiliary diagnostic method for the death of sepsis patients’.

## Data Availability

All data generated or analysed during this study are included in this published article.

## Abbreviations

APACHE2: Acute Physiology and Chronic Health Evaluation
CRP: C-reactive protein
ICU: Intensive Care Unit
NLR: neutrophil-to-lymphocyte ratio
MLR: monocyte-to-lymphocyte ratio
MPV/PC: mean platelet volume to platelet count
PCT: Procalcitonin
PLR: platelet-to-lymphocyte ratio
SOFA: Sequential Organ Failure Assessment
